# Cancer symptom cluster research in pediatric oncology: a work in progress

**DOI:** 10.37349/etat.2024.00225

**Published:** 2024-04-24

**Authors:** Luciana Chain Veronez, Luís Carlos Lopes-Júnior

**Affiliations:** Istituto Nazionale Tumori-IRCCS-Fondazione G. Pascale, Italy; ^1^Department of Childcare and Pediatrics, Ribeirão Preto Medical School, University of São Paulo (USP), Ribeirão Preto, SP 14040-902, Brazil; ^2^Health Sciences Center, Federal University of Espírito Santo (UFES), Vitoria, ES 29043-900, Brazil

**Keywords:** Cancer symptom clusters, pediatric oncology, translational research

## Abstract

In the 21st century, advances in basic research have provided new insights in the field of pediatric oncology. Pediatric patients tend to experience higher levels of distressing symptoms, which together form a symptom cluster. In clinical practice, these symptom clusters are reported daily by children and adolescents with cancer. Translational research has emerged as the translation of new knowledge from basic science into clinical practice. Understanding how neuroimmunoendocrine pathways regulate cancer development and the aspects underlying the specific therapies, such as chemotherapy and immunotherapy, is an important frontier for future research in pediatric oncology. The goal of translational research is to show how different variables in tumor and patient characteristics explain the differential effects of interventions, as translational research provides new insights into the management of cancer symptoms in children and adolescents with cancer. Together, this approach could lead to improvements in pediatric oncology care worldwide.

## Introduction

Cancer is still a contemporary global public health problem and the second leading cause of mortality in many countries [1]. Pediatric neoplasms represent a group of diseases with specific characteristics, mainly related to histopathology and clinical behavior. In fact, most of the neoplasms affecting this population have a short latency period and are more aggressive, but often with a better response to treatment and consequently a better prognosis [2], especially when the diagnosis is made early and the management is carried out by specialized, multidisciplinary and interdisciplinary teams [3].

Recent global estimates suggest approximately 400 thousand new cases of cancer in children and adolescents aged 0–19 years, with approximately 90% occurring in low- and middle-income countries [4–6]. Although pediatric neoplasms are relatively rare, accounting for 1–4% of all childhood malignancies [5], in developing countries they account for 3–10% of all neoplasms [6]. Annually, more than 15,000 new cases of cancer are diagnosed in children and adolescents, resulting in 1,960 deaths [2, 5]. Although advances in cancer treatment over the past four decades have remarkably improved the 5-year survival rate for children from 10% to approximately 80% [1, 5], the incidence of childhood cancer has continued to rise [2, 5].

Compared with the adult cancer population, pediatric patients tend to experience higher levels of discomfort symptoms [7–16]. Some of these symptoms appear to be related to each other, forming a symptom cluster [17]. Cancer symptom clusters (CSCs) comprise a set of symptoms that are related to each other, which may create a synergistic effect between them and may be predictable [17]. Although previously reported as three or more concurrent symptoms related to each other [17], the definition of a symptom cluster has been revised to correspond to symptoms that are clinically meaningful and related to each other, and with significant variance in the cluster [18, 19]. Current evidence suggests that inflammation may be a key event in the development of CSCs [20–22]. The release of proinflammatory cytokines due to cancer treatment has been shown to be associated with subsequent cognitive impairment [21–24]. For example, researchers have indicated that peripheral blood interleukin-1β (IL-1β), IL-6, tumor necrosis factor-α (TNF-α), and C-reactive protein are the most reliable biomarkers of inflammation in patients with depression [25]. Overall, the etiology of CSC is very complex, with symptoms in these clusters exacerbating each other, leading to a reduction in patients’ health status and quality of life [26–29]. In addition, common molecular mechanisms may underlie the interaction between the immune, endocrine, and central nervous systems that orchestrate a series of responses capable of installing physiological and biobehavioral changes that result in these clusters [20, 22]. Many of the patients may live with these unpleasant symptoms for many years, even after the end of cancer treatment [30]. Therefore, the adoption of procedures and treatments that provide relief from physical and emotional symptoms, as well as emotional support for pediatric patients, is critical in cancer care [12, 13, 16, 31].

## CSC in pediatric oncology: state of the art

Experimental studies have reported a neuropsychological symptom cluster in animals exposed to infectious conditions and treated with pro-inflammatory cytokines. As a result of these inflammatory conditions, the “sickness behavior” was established—a phenomenon that refers to the set of behavioral changes that are accompanied by numerous pathological events that appear to occur without any pathophysiological link [32]. Sickness behavior was first reported in a study evaluating the effects of bacterial products on behavior. The effects of cytokines such as IL-6, IL-1, and TNF-α, whose inflammatory roles have been extensively reported [33–35], were associated with sickness behavior.

**Figure 1:**
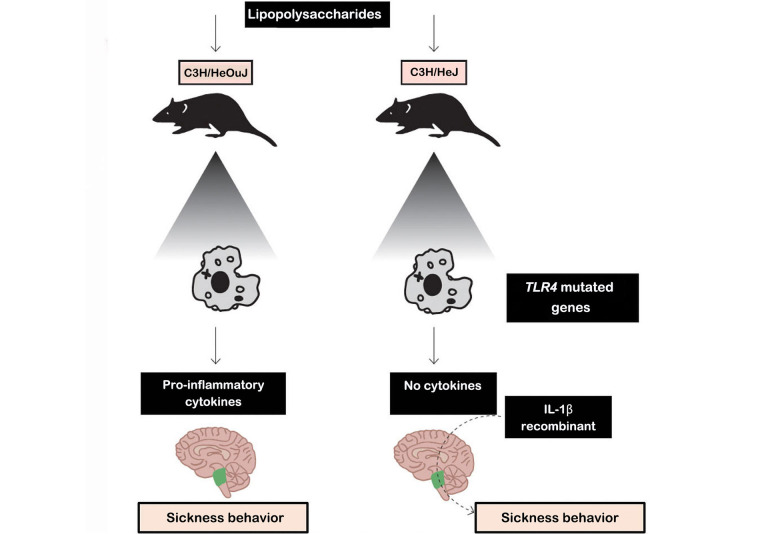
Experiment showing the behavior of the disease. C3H/HeJ mice: commonly called C3H, are used as a general purpose strain in a wide variety of research areas including cancer. C3H/HeJ mice are refractory to lipolysaccharides. C3H/HeOuJ mice: are used as a general purpose strain in a wide variety of research areas including cancer and sensorineural, research *Note.* Adapted with permission from “The concept of sickness behavior: a brief chronological account of four key discoveries,” by Johnson RM. Vet Immunol Immunopathol. 2002;87:443–50 (https://doi.org/10.1016/s0165-2427(02)00069-7). © 2024 Elsevier B.V.

In Figure 1, two types of mice were used [control mice and Toll-like receptor 4 (TLR4) knockout mice, which do not synthesize TLR4 receptors], which are responsible for the synthesis of IL-1β. The results of this experiment showed that macrophages from normal mice when stimulated with lipopolysaccharides (LPS), secrete cytokines (especially IL-1β). Cytokines transmit messages from the periphery to the brain via humoral and neural pathways [34].

In fact, peripheral cytokines may not enter the brain themselves but instead induce the expression of other cytokines in the brain that cause disease behavior. Furthermore, inflammatory stimuli in the periphery (e.g., LPS and pro-inflammatory cytokines) induce transcripts for IL-1β, IL-6, and TNF-α in discrete brain areas of the mouse. Thus, inflammatory stimuli in the periphery induce perivascular microglial cells to express cytokines. In addition, cytokines in the periphery can send a message to the brain via the vagus nerve. When activated by peripheral cytokines, the vagus can activate specific neural pathways involved in disease behavior. However, vagal activation also appears to stimulate microglia in the brain to produce cytokines [34]. Thus, just as the brain uses humoral and neural pathways to influence the immune system, the immune system also uses humoral and neural pathways to influence the brain and behavior.

Both animal and human studies have shown that cytokine infusion also induces sickness behavior. In humans, the phenomenon of sickness behavior, including symptoms such as insomnia, pain, fatigue, depression, and cognitive dysfunction, has also been observed in cancer patients and is associated with high levels of pro-inflammatory cytokines [22, 34]. Indeed, common biological mechanisms may underlie the interaction between the endocrine, nervous, and immune systems, which together orchestrate a series of responses capable of installing behavioral and physiological changes in animals and humans [33, 34]. Studies on sickness behavior and CSC in oncological patients support the hypothesis that proinflammatory cytokines are associated with the biological mechanisms underlying the occurrence of these symptomatic groups [33–35]. For example, the release of cytokines such as IL-12p70, TNF-α, IL-1β, IL-8, IL-6, and interferon-γ (IFN-γ) leads to CSC, including cancer-related fatigue (CRF), sleep disturbances, pain, anxiety, and depression. Alterations in cytokine levels and other neuroimmunologic processes may be central players in the production of symptoms, as well as potentially related to their prevention and treatment [10, 22, 35–37].

A previous study compared the levels of the cytokines IL-12p70 and IL-10 in 59 children (aged 2–15 years) with soft tissue sarcoma and showed that those in remission had increased IL-12 production and decreased IL-10 levels. The opposite pattern was shown in patients who relapsed, with increased levels of IL-10 and decreased production of IL-12 [38]. Furthermore, a pilot study investigating the feasibility of biomarkers related to CRF and stress in pediatric osteosarcoma inpatients receiving chemotherapy showed an overall reduced trend in cortisol levels over time after non-pharmacological intervention. A similar response pattern was observed for TNF-α levels. In addition, pediatric patients with metastatic osteosarcoma showed a linear trend for decreased levels of matrix metalloproteinase-9 (MMP-9) after receiving non-pharmacologic intervention [39].

## Future perspectives

There is great potential for advancing symptom science through research that evaluates the psychoimmunoendocrine pathways involved in the genesis of CSC. Once the association or level of sensitivity and specificity of biomarkers has been identified, they can be used to determine the response to interventions, leading to an accurate determination of their efficacy and/or for whom and in what context the interventions will work, that is, what dose, type, how often, when, and for how long they should be used [10, 12, 40].

In line with several precision health initiatives [41–43], one of the major challenges in pediatric oncology research is the identification of molecular or phenotypic biomarkers to discriminate individuals at high or low risk for symptom burden, or even novel targets and effective supportive care interventions that may improve patient outcomes [44]. In this context, the development of optimal approaches capable of identifying mechanisms of individual variability in symptom experience requires a detailed understanding of the factors associated with higher symptom burden. To this end, individual biosignatures that integrate both genomic and phenotypic components, such as integrative “omics” (genomics, metabolomics, transcriptomics, epigenomics, and proteomics) assessments, as well as environmental, lifestyle, and psychosocial factors, may aid in this process [45]. Indeed, recent research has shown that the use of reliable and valid self-report measures combined with various biomarkers has provided important information about the mechanisms underlying symptom experience [46, 47].

In addition, the identification of phenotypic differences between pediatric patients with stable symptoms and those whose symptoms change over time is of great interest to provide better personalized patient care. Understanding the characteristics and symptoms of pediatric cancer patients will improve the prediction of cases at highest or the lowest risk of developing adverse CSC, allowing clinicians to define management approaches and target intensive symptom screening. Such predictive factors will also improve symptom outcomes once they can help anticipate patient and family education and other preventive interventions for each individual patient. However, there is still a knowledge gap regarding the phenotypes of symptom burden in the pediatric cancer survivor population [44].

The expert panel proceedings and recommendations for advancing the science of symptom management through symptom clusters research [48], have identified 5 key critical points to be addressed in this field: (I) to define the characteristics of CSCs; (II) to prioritize CSCs as well as their underlying mechanisms; (III) to adequately and reliably measure CSCs; (IV) to evaluate the effective targeted interventions for CSC management; (V) to propose new advanced analytical techniques to better assess CSCs in research [48] (Table 1).

**Table 1:** Directions for future CSC research in pediatric oncology based on the expert panel proceedings and recommendations for advancing the science of symptom management through symptom clusters research [48]

**Key area**	**Some directions**
To define the characteristics of CSCs	Establish a common conceptual framework for evaluating the measurement of CSCs. Determine the specific characteristics to define a CSC and develop an operational definition for a CSC in pediatric oncology. Identify the sentinel symptom within a CSC. Evaluate the relationship between signs (objective indications) and symptoms (subjective sensations) within a CSC. Evaluate for CSC in defined patient subgroups in pediatric oncology. Develop qualitative approaches to identify and prioritize CSCs according to their importance to patients. Develop a consistent approach to identify patient subgroups based on a pre-specified CSC. Identify the most common *de novo* CSCs in pediatric oncology. Replicate studies of subgroups of pediatric cancer patients with similar and different experiences with a prespecified CSC; tailor assessment, interventions, and outcome measures to the evolving CSC over the course of childhood cancer. Determine the phenotypic as well as molecular predictors and/or risk factors for the development of prespecified CSC in pediatric cancer patients under different types of antineoplastic therapies. Evaluate the potential for using large data sets and the electronic health record to assess CSC. Develop and test methods to assess CSC in pediatric cancer patients who cannot self-report symptoms. Develop and test methods to assess CSC using surrogates for CSC reporting (e.g., physicians, nurses, family caregivers, informal caregivers).
To prioritize CSCs and their underlying its mechanisms	Develop a core set of symptom inventories for CSC research in pediatric oncology. Evaluate the molecular mechanisms underlying CSC, including (I) inflammation/immune system, (II) hypothalamic-pituitary-adrenal axis activation, (III) sympathetic nervous system activation, (IV) central nervous system alterations, (V) circadian rhythm alterations, etc. Determine the best approaches to evaluate the underlying genetic and epigenetic mechanisms for CSC in pediatric oncology. Determine the best methods to assess the biobehavioral mechanisms for CSC in pediatric oncology. Develop and evaluate animal models of CSC in pediatric oncology. Determine whether common mechanisms exist for CSC in pediatric oncology. Develop a systematic approach for biomarker selection for CSC research in pediatric oncology.
Appropriately and reliably measure the CSCs	Use qualitative methods to identify common and disease-specific CSC across the pediatric cancer spectrum. Develop generic and disease-specific measures to assess CSC in pediatric oncology. Determine the optimal approach to data collection for CSC research in pediatric oncology. Compare and contrast the number and types of CSCs, as well as changes over time, identified in pediatric cancer patients using a variety of analytical techniques. Evaluate the validity, reliability, and responsiveness of PROMIS measures in pediatric oncology CSC research. Establish a common core data set for pooling data and assessing data comparability across pediatric oncology CSC studies. Use new methods to refine measures for CSC (e.g., Rasch analysis). Establish red-flag values for CSC that warrant intervention(s) for pediatric cancer patients. Correlate various outcomes of CSC (e.g., functional status, social status, quality of life, mortality, survival, costs, health care utilization, patient satisfaction, and caregiver burden).
To evaluate the effective targeted interventions for CSC management	Evaluate the use of new trial designs to determine if they can be used to tailor interventions to treat single or multiple symptoms within a CSC in pediatric oncology. Determine the most effective interventions for different pediatric oncology CSCs. Determine the most appropriate outcome for a pediatric oncology CSC intervention trial. Evaluate the use of technology in pediatric oncology CSC research.
To propose new advanced analytical techniques for better assessment of CSCs in research	Applying new analytical techniques to pediatric oncology CSC research (e.g., latent transition analysis, evolutionary algorithms, machine learning, and risk stratification). Establish guidelines for the selection of optimal analytic strategies for CSC research in pediatric oncology.

PROMIS: Patient-Reported Outcomes Measuremen Information System

*Note.* Adapted with permission from “Advancing symptom science through symptom cluster research: expert panel proceedings and recommendations,” by Miaskowski C, Barsevick A, Berger A, Casagrande R, Grady PA, Jacobsen P, et al. J Natl Cancer Inst. 2017;109:djw253 (https://doi.org/10.1093/jnci/djw253). © 2024 Oxford University Press.

In recent decades, important advances in basic research have provided new knowledge in many different fields, including pediatric oncology [49]. In this context, translational research has emerged as the translation of new knowledge from basic science to clinical practice [49], which can also have a bidirectional flow with complex feedback relationships commonly referred to as the bench, bedside, and back again (3 Bs) [10, 50].

Immunological biomarkers, for example, have been recognized as important markers for the study of CSC, with a primary focus on cytokines [37]. Although numerous biomarkers have been reported for cancer management, their use to assess CSC and quality of life in patients undergoing cancer treatment is still in its infancy in pediatric oncology [8, 10, 48, 50–52]. Understanding how neuroimmunoendocrine pathways regulate cancer development and the aspects underlying specific therapies, such as chemotherapy and immunotherapy, is an important frontier for future research in pediatric oncology [10, 48, 53, 54].

Recent approaches to translational research in pediatric oncology have revealed a wide field of analysis for understanding the relationships between different systems and the bio-behavioral changes in pediatric cancer patients. It should be emphasized that in clinical practice, clusters of symptoms are reported daily by children and adolescents with cancer. Therefore, healthcare professionals have a key role in educating patients about the risk of CSCs based on each individual’s genomic profile. With advances in personalized cancer care, understanding individual susceptibilities to symptoms and whether a “driving” symptom triggers or may influence other symptoms within a cluster is of great interest. The goal of translational research is to understand how a set of variables explains different effects of interventions in each individual, once it provides new insights for cancer symptom management in children and adolescents with cancer, which could lead to improvements in pediatric oncology care around the world.

## Abbreviations

CSCs: cancer symptom clusters
IL-1β: interleukin-1β
TNF-α: tumor necrosis factor-α

## Declarations

### Copyright

© The Author(s) 2024.
